# Text-based nutrient estimation by large language models: a comparative evaluation against the Chinese Food Composition Table

**DOI:** 10.3389/fnut.2026.1952757

**Published:** 2026-10-05

**Authors:** Yongwen Cao, Zhendong Liu, Ning Wang, Min Chen, Hongyan Wang

**Affiliations:** 1 Gansu Provincial Maternal and Child Health Care Hospital (Gansu Provincial Central Hospital), Lanzhou, Gansu Province, China; 2 Gansu University of Chinese Medicine, Lanzhou, Gansu Province, China

**Keywords:** artificial intelligence, Chinese foods, food composition database, large language models, nutrient estimation

## Abstract

**Background:**

Large language models (LLMs) increasingly provide nutrient information, but their accuracy for Chinese foods and their consistency across repeated queries are not well established.

**Objective:**

To compare nutrient estimates from nine general-purpose LLMs with the Chinese Food Composition Table (CFCT) and assess within-model reproducibility.

**Methods:**

For 299 commonly consumed Chinese foods, each model estimated energy and 14 nutrients per 100 g in three independent runs (8,073 food-level outputs). Model means were compared with CFCT values using absolute-agreement intraclass correlation coefficients (ICCs), bias, mean absolute error (MAE), root mean squared error, percentage error, and Bland–Altman limits of agreement. Repeated-run ICCs and coefficients of variation assessed reproducibility.

**Results:**

Agreement was excellent for energy, fat, carbohydrate, sodium, and vitamin C across all nine models, and good to excellent for protein. Performance was less consistent for vitamin A, zinc, iron, and potassium. Repeated-run stability was high for energy and macronutrients but lowest for vitamin A. GLM had the most favorable combined accuracy-stability profile, followed by Minimax and Qwen. Even when aggregate agreement was high, individual food estimates sometimes showed substantial error.

**Conclusion:**

General-purpose LLMs can approximate energy and macronutrient values for Chinese foods under controlled text-based conditions, but micronutrient estimates remain less dependable. Values intended for clinical or other high-stakes applications should be verified using validated food-composition resources and, where appropriate, qualified nutrition professionals.

## 1 Introduction

Accurate dietary assessment is central to nutrition research and clinical practice, yet conventional methods such as 24-h recalls, food-frequency questionnaires, and weighed food records remain vulnerable to recall error, portion-size misestimation, respondent burden, and incomplete reporting. Digital tools can reduce some of this burden, but their usefulness still depends on reliable food identification and nutrient estimation (1). Accurate nutrient information carries particular weight in China, where national burden-of-disease estimates identify dietary iron deficiency and vitamin A deficiency as leading contributors to nutritional-deficiency-related disability, concentrated among young children and older adults (2), and nationally representative dietary-intake data show that a substantial proportion of Chinese children do not meet estimated average requirements for iron, zinc, and vitamin A (3). At the same time, consumers and patients are increasingly using generative AI tools to seek health and nutrition information, making the reliability of the nutrient values returned by these systems a practical as well as methodological concern (4).

Large language models (LLMs) offer a different route because they can generate nutrient estimates directly from natural-language food descriptions and, in multimodal systems, from images. Without retrieval from a validated nutrition database, however, these values are generated probabilistically rather than obtained through deterministic database lookup. Image-based evaluations have reported substantial variation across models, foods, and nutrients, particularly when portion size, preparation method, sodium, fortification, or hidden ingredients must be inferred (5–10). These real-world tasks combine several sources of uncertainty, making it difficult to separate failures of food recognition or portion estimation from limitations in the model’s underlying nutrient knowledge.

Text-based studies provide a more controlled test of that nutrient-knowledge component, but the evidence base is still developing. Haman et al. reported nutrient- and food-specific variation in ChatGPT estimates for 236 foods (11). Early clinical-focused evaluations similarly found inconsistent performance when general-purpose chatbots were applied to specialized dietary counseling, such as renal diet support (12). Lawabni et al. subsequently evaluated four LLMs across 194 frequently consumed foods in the United States and found strong agreement for energy and most macronutrients but weaker performance for several micronutrients (13). Repeated-query work using commercial ready-to-eat foods has further shown that apparent accuracy and within-model consistency can vary by nutrient and model, indicating that a single response may not adequately characterize model behavior (14). Together, these studies establish that text-based nutrient estimation is feasible to benchmark, but they do not establish clinical interchangeability with reference data and leave important questions about geographic food contexts, model diversity, and reproducibility.

The present study was therefore designed as a controlled computational benchmark rather than a simulation of routine consumer or clinical use. Its incremental contribution is not the general demonstration that LLMs can generate nutrient estimates from text, but the systematic evaluation of a large set of Chinese foods using a Chinese reference standard, nine contemporary Chinese and international general-purpose LLMs, 15 dietary components, and three fully independent runs. Chinese foods encompass diverse ingredients, preparation traditions, and regional variants, while several widely used Chinese-developed LLMs have received little systematic evaluation in nutrition research. Domain-specific food systems and retrieval-augmented approaches also suggest that structured food knowledge can materially affect performance (15–20). We therefore compared model estimates with the Chinese Food Composition Table, 6th edition, for 299 commonly consumed Chinese foods (19). The primary objective was to quantify agreement and error; secondary objectives were to compare models, assess within-model reproducibility, and examine food-group differences. We expected stronger performance for energy and macronutrients than for micronutrients.

## 2 Methods

### 2.1 Study design and reference data

This computational benchmarking study compared LLM-generated dietary-component estimates with CFCT reference values. This study was reported in accordance with the TRIPOD-LLM reporting guideline for studies evaluating large language models. The unit of analysis was a food item, standardized to a 100-g edible portion. The source database contained 1,745 food records. Infant foods, alcoholic beverages, other/medicinal-food categories, and clearly rare, branded, or anomalous items were excluded. Quotas were specified across the major food categories, and stratified purposive sampling considered common Chinese-food keywords, generic naming, edible-portion information, and name specificity. This process yielded 300 candidate foods. One duplicated standardized query name was then removed, leaving 299 unique foods across 18 food groups. The food-group composition of the final sample is provided in Supplementary Table S1.

For each food, we extracted energy (kcal) and 14 nutrients from the CFCT: protein (g), total fat (g), carbohydrate (g), dietary fiber (g), sodium (mg), potassium (mg), calcium (mg), iron (mg), zinc (mg), vitamin A (μg retinol equivalents [RE]), vitamin C (mg), thiamin (mg), riboflavin (mg), and niacin (mg). Each standardized query name was linked to its reference record by a predefined unique food identifier rather than by unrestricted text matching.

### 2.2 Models, prompting, and quality control

Nine LLMs available in July 2026 were evaluated, including models from Chinese and international developers (Table 1). Models were accessed through official or OpenAI-compatible application programming interfaces (APIs). No web search, external retrieval system, or food-composition database was supplied during inference.

**Table 1:** Large language models evaluated in the study.

Model	Full model identifier	API access route	**Temperature**
DeepSeek	Deepseek-v4-flash	DeepSeek API	0.2
Qwen	Qwen3.7-plus	Alibaba Cloud DashScope API	0.2
GLM	Glm-5.1	Zhipu AI API	0.2
Minimax	Minimax-M3	MiniMax API	0.2
Kimi	Kimi-k2.7-code-highspeed	Moonshot AI API	1.0*
Claude	Claude-sonnet-4-5	Anthropic API	0.2
Mistral	Mistralai/mistral-large-3-675b-instruct-2512	NVIDIA Build	0.2
Gemini	Gemini-3.1-flash-lite	Google Gemini API	0.2
ChatGPT	Gpt-5.2	OpenAI API	0.2

*The selected Kimi endpoint accepted only a temperature of 1.0; all other models were queried at 0.2.

All models received the same food names, dietary-component definitions, units, ordering, and output constraints. The prompt asked the model to estimate each component per 100 g edible portion and return structured numeric data only, preserving every food name exactly. Foods were submitted in batches of 20. Except where an API imposed a different setting, temperature was 0.2; the selected Kimi endpoint required 1.0.

Each model completed three fully independent runs of all 299 foods, with no conversational state carried over between runs. The final run-level dataset contained 8,073 records (299 foods × 9 models × 3 runs). Responses were accepted only if they were parseable, contained all expected foods, preserved the submitted names, and supplied numeric values for all 15 dietary components. Food-name values were matched exactly to the prespecified standardized query names; no string normalization, case-folding, whitespace trimming, fuzzy matching, or manual name correction was applied to food-name values. An initial DeepSeek run contained nine missing energy values within one batch; that incomplete run was discarded and repeated in full, and the complete replacement run was retained. After quality control, all 27 model-run combinations contained 299 foods with complete component estimates, no records were lost because of name-matching failure, and no dietary-component value was manually edited (Supplementary Table S2). For the primary accuracy analysis, the three estimates for each model-food-component combination were averaged, yielding 2,691 model-food records. The original run-level values were retained for reproducibility and sensitivity analyses.

### 2.3 Statistical analysis

For each model and dietary component, agreement between the three-run mean estimate and the CFCT value was assessed across 299 foods using a two-way random-effects intraclass correlation coefficient for absolute agreement and single measurements, ICC(A,1). The three-run aggregate was treated as one model-derived measurement per food; ICC (A,1) was therefore retained as the primary agreement statistic for the model-derived estimate versus the single CFCT reference value. Average-measures ICC(A,k), Pearson correlation, and identity-line R^2^ were calculated as secondary measures. ICCs were interpreted as excellent (≥0.90), good (0.75 to <0.90), moderate (0.50 to <0.75), or poor (<0.50) (21).

Error was summarized using mean bias (model minus reference), mean absolute error (MAE), root mean squared error (RMSE), mean absolute percentage error (MAPE), and median absolute percentage error. Foods with a reference value of zero were excluded only from percentage-error calculations because percentage error is undefined at zero; zero-valued foods remained in ICC, bias, MAE, and RMSE calculations. Because small positive denominators can also inflate percentage errors, the number of zero and nonzero reference values was tabulated for each component, and MAPE was interpreted alongside absolute-error and agreement measures. Nonparametric 95% confidence intervals for bias, MAE, RMSE, and MAPE were obtained from 1,000 food-level bootstrap samples.

Bland–Altman analysis quantified mean differences and 95% limits of agreement (LoA) for each model-component combination. Reproducibility was assessed from the three original runs using repeated-measures ICC(A,1), ICC(A,k), and food-level coefficients of variation (CVs). CV was undefined when the three-run mean was zero. Accuracy metrics were also calculated within food groups as descriptive subgroup analyses; these analyses were interpreted cautiously because food-group sample sizes were unequal.

An exploratory composite ranking summarized accuracy and reproducibility. Within each dietary component, models were ranked on six equally weighted criteria: accuracy ICC(A,1), MAE, RMSE, MAPE, repeated-run ICC(A,1), and median repeated-run CV. Component-specific ranks were averaged across all 15 dietary components; lower scores indicated better combined performance. Because the score combines metrics with different statistical properties, it was treated as a descriptive benchmark rather than a clinical index. Additional sensitivity analyses were conducted to assess robustness. First, the three-run median replaced the three-run mean to assess whether isolated extreme run-level estimates influenced accuracy metrics or model ordering. Second, the robustness of percentage-error findings and the composite ranking was explored by (i) replacing MAPE with median absolute percentage error, (ii) omitting the percentage-error criterion, and (iii) recalculating MAPE after excluding positive CFCT reference values at or below the component-specific 5th percentile. The last analysis was exploratory and was used specifically to assess leverage from near-zero denominators. Analyses were performed in Python 3.14 using pandas and NumPy.

### 2.4 Ethics statement

The study used published food-composition data and model-generated outputs only. It involved no human participants, patient records, personal identifiers, biological specimens, or clinical interventions; institutional review board approval and informed consent were therefore not required.

## 3 Results

### 3.1 Dataset and overall model performance

The final dataset contained 299 unique foods across 18 CFCT food groups. Group sizes ranged from 4 to 45 foods; the largest groups were vegetables and vegetable products (*n* = 45), cereals and cereal products (*n* = 35), fruits and fruit products (*n* = 30), livestock meat and products (*n* = 30), and fish, shrimp, crab, and shellfish (*n* = 25) (Supplementary Table S1). All nine models contributed three complete independent runs after quality control, giving 8,073 run-level records. The final dataset contained no duplicate model-run-food keys, missing food identifiers, food-name matching failures, missing LLM component values, or missing reference component values. The only failed validation involved an initial DeepSeek run with nine missing energy values in one batch; the entire run was repeated and the complete replacement run was used in all analyses (Supplementary Table S2).

GLM ranked first overall in the exploratory composite score (1.57), with the highest mean accuracy ICC (0.906) and mean stability ICC (0.990). Minimax ranked second and Qwen third. Claude and DeepSeek were intermediate, whereas Gemini, Mistral, Kimi, and ChatGPT ranked lower under the tested versions, prompts, API settings, dietary components, and reference dataset (Table 2).

**Table 2:** Overall model ranking based on combined accuracy and repeated-run stability.

Rank	Model	Overall rank score	Mean accuracy ICC	Mean MAPE (%)	Mean stability ICC
1	GLM	1.57	0.906	61.76	0.990
2	Minimax	3.73	0.852	60.31	0.955
3	Qwen	4.19	0.818	70.09	0.932
4	Claude	5.16	0.839	72.54	0.910
5	DeepSeek	5.46	0.831	66.03	0.911
6	Gemini	5.57	0.716	78.08	0.982
7	Mistral	5.78	0.738	82.63	0.959
8	Kimi	5.97	0.831	78.56	0.894
9	ChatGPT	7.56	0.711	90.28	0.964

The overall ordering of all nine models was unchanged when the three-run median was used instead of the mean. GLM remained first under every alternative composite specification examined. Replacing MAPE with median absolute percentage error produced only minor lower-rank changes, whereas omitting the percentage-error criterion changed the ordering below first place, with Qwen moving to second and Minimax to third. Using the near-zero-trimmed MAPE preserved the original top five models and changed only the relative positions of Mistral and Gemini. These findings support interpreting the composite ranking as descriptive rather than as a fixed hierarchy of model quality (Supplementary Tables S4, S5).

### 3.2 Agreement and error by dietary component

Agreement with the CFCT depended strongly on dietary component. Mean ICCs across models were highest for sodium (0.984), fat (0.978), energy (0.971), carbohydrate (0.969), vitamin C (0.945), calcium (0.914), and protein (0.908). Every model achieved excellent agreement for energy, carbohydrate, fat, sodium, and vitamin C; protein was good or excellent in all models. Dietary fiber was also generally strong (mean ICC, 0.862).

Micronutrient performance was less consistent. Mean ICCs were 0.796 for thiamin, 0.721 for riboflavin, 0.715 for niacin, 0.697 for potassium, 0.599 for iron, 0.527 for zinc, and 0.486 for vitamin A. Vitamin A was the weakest nutrient overall: only GLM reached good agreement (ICC = 0.786), and four models were in the poor range. Potassium varied from excellent agreement for GLM (0.924) to poor agreement for ChatGPT (0.351), Gemini (0.399), and Mistral (0.447). Zinc remained difficult for every model, with a maximum ICC of 0.633 (Table 3).

**Table 3:** Dietary-component-specific agreement across the nine models.

Dietary component	Mean ICC	Min-Max ICC	E/G/M/P*
Energy (kcal)	0.971	0.945–0.989	9/0/0/0
Carbohydrate (g)	0.969	0.940–0.993	9/0/0/0
Protein (g)	0.908	0.839–0.972	6/3/0/0
Fat (g)	0.978	0.965–0.989	9/0/0/0
Dietary fiber (g)	0.862	0.702–0.967	5/1/3/0
Sodium (mg)	0.984	0.979–0.989	9/0/0/0
Potassium (mg)	0.697	0.351–0.924	1/5/0/3
Calcium (mg)	0.914	0.854–0.976	6/3/0/0
Iron (mg)	0.599	0.332–0.910	1/1/4/3
Zinc (mg)	0.527	0.486–0.633	0/0/7/2
Vitamin A (μg RE)	0.486	0.224–0.786	0/1/4/4
Vitamin C (mg)	0.945	0.902–0.967	9/0/0/0
Thiamin (mg)	0.796	0.588–0.905	1/6/2/0
Riboflavin (mg)	0.721	0.603–0.836	0/3/6/0
Niacin (mg)	0.715	0.605–0.789	0/3/6/0

*E/G/M/P, number of models with excellent/good/moderate/poor agreement according to prespecified ICC thresholds.

Absolute errors for energy and macronutrients were smallest for GLM and Minimax. Energy ICCs ranged from 0.945 to 0.989, with MAEs of 9.85 kcal for GLM and 10.24 kcal for Minimax. Protein ICCs ranged from 0.839 to 0.972; the lowest protein MAEs were 0.83 g for GLM and 0.86 g for Minimax. The corresponding lowest MAEs were 0.93–1.01 g for fat and 1.59–1.86 g for carbohydrate. Reference zeros were unevenly distributed across dietary components: vitamin C had 204/299 zero values (68.2%), sodium 153/299 (51.2%), dietary fiber 144/299 (48.2%), and vitamin A 117/299 (39.1%), leaving 95, 146, 155, and 182 foods, respectively, for MAPE calculations. Percentage errors were therefore interpreted alongside absolute error and ICC (Supplementary Table S3).

The exploratory near-zero sensitivity analysis confirmed substantial denominator effects for several components. After excluding positive CFCT values at or below the component-specific 5th percentile, the mean MAPE across models decreased from 242.4 to 106.2% for sodium, from 145.8 to 83.7% for vitamin A, from 74.9 to 49.3% for dietary fiber, and from 83.8 to 65.6% for vitamin C. By contrast, energy MAPE changed only modestly, from 13.4 to 12.6%. These findings indicate that very small reference values materially inflated percentage-based errors for selected components but did not affect absolute-error or ICC calculations (Supplementary Table S3).

Bland–Altman analysis showed small average bias for the three highest-ranked models but wider food-level LoA. For energy, mean differences were −1.01 kcal/100 g for GLM, −0.34 for Minimax, and −4.88 for Qwen, with 95% LoA of −57.76 to 55.74, −54.19 to 53.52, and −72.93 to 63.17 kcal/100 g, respectively. Protein, fat, and carbohydrate mean differences were within about 1.5 g/100 g for these models, but the LoA again indicated potentially meaningful error for individual foods.

### 3.3 Repeated-run reproducibility and food-group patterns

Repeated-run stability was high for energy (mean ICC, 0.992), fat (0.996), carbohydrate (0.989), protein (0.981), and sodium (0.997). Vitamin A was the least reproducible dietary component (mean ICC, 0.778), followed by iron, riboflavin, and potassium. At the model level, GLM had the highest mean stability ICC (0.990) and essentially zero median within-food CV across components. Gemini, ChatGPT, Mistral, and Minimax also showed high mean stability, whereas Kimi, Claude, and DeepSeek were lower. Qwen’s minimum stability ICC (0.236) reflected poor repeatability for vitamin A (Table 4). When the median of the three runs replaced the mean in the accuracy analysis, the overall model ordering was unchanged. Only 6 of 135 model-component combinations (4.4%) crossed a prespecified ICC category threshold; two of these involved vitamin A changing from moderate to poor agreement (DeepSeek, ICC 0.650 to 0.467; Minimax, 0.504 to 0.443). The sensitivity analysis therefore supported the model-level conclusions while reinforcing the instability of selected micronutrient estimates (Supplementary Tables S4, S6).

**Table 4:** Within-model stability across three repeated runs.

Model	Mean stability ICC	Minimum stability ICC	Mean median CV (%)
GLM	0.990	0.969	0.00
Minimax	0.955	0.881	10.33
Qwen	0.932	0.236	2.40
Claude	0.910	0.645	2.10
DeepSeek	0.911	0.600	17.48
Gemini	0.982	0.909	1.19
Mistral	0.959	0.759	0.84
Kimi	0.894	0.669	9.80
ChatGPT	0.964	0.870	8.21

Errors also varied by food group. Percentage errors for energy were lower in fats and oils, sugar and honey products, instant foods, nuts and seeds, and cereals, and higher in condiments, beverages, fungi and algae, and several meat and poultry groups. Groups with component values near zero often had inflated percentage errors despite modest absolute differences. Beverages, animal-source foods, snack foods, and condiments showed larger errors for selected components. These subgroup patterns were descriptive because group sizes differed substantially (*n* = 4–45) and the underlying component distributions were heterogeneous.

## 4 Discussion

In this benchmark of nine general-purpose LLMs, aggregate agreement with CFCT values was high for energy and several macronutrients, whereas performance for vitamin A, zinc, iron, and potassium was substantially less consistent. Repeated querying showed high stability for energy and macronutrients but greater variability for selected micronutrients. GLM had the most favorable combined accuracy-reproducibility profile under the tested conditions. These findings support two distinct conclusions: model performance depends strongly on the dietary component being estimated, and reproducibility cannot be inferred from accuracy, or accuracy from reproducibility.

The overall pattern is consistent with previous text-based work but extends it in several specific ways. Lawabni et al. reported high agreement for energy and most macronutrients in 194 US foods, with greater variability for several micronutrients, while Haman et al. found nutrient- and food-specific differences in ChatGPT estimates from food names alone (11, 13). Repeated-query evaluation of commercial ready-to-eat foods has also shown nutrient-dependent variation across chatbots (14). The present study adds a larger Chinese-food benchmark, a Chinese reference standard, five Chinese-developed models, 15 dietary components, and three complete independent runs. Accordingly, the principal novelty lies in the breadth and geographic context of the benchmark and in the explicit assessment of within-model reproducibility, rather than in showing for the first time that LLMs can generate nutrient estimates from text.

High aggregate ICCs should nevertheless be interpreted cautiously. ICC summarizes agreement across a set of foods and does not establish that the error for an individual food is small enough for a particular clinical or practical purpose. This distinction is visible in our Bland–Altman results: GLM and Minimax had energy biases near zero, yet their 95% limits of agreement extended to approximately +/−55 kcal/100 g, and Qwen’s limits were wider. Recent work on LLM-assisted carbohydrate counting illustrates why clinically meaningful thresholds matter: when formal equivalence testing was applied against clinician-derived reference values, only one of four tested models met a prespecified +/−5% equivalence criterion, despite generally favorable correlations and small average biases (22). Our study did not prespecify clinical equivalence margins, and its ICCs, MAEs, and Bland–Altman results therefore describe benchmark performance rather than clinical equivalence.

The practical implications should be framed accordingly. By supplying a standardized food name and a fixed 100-g edible portion, we deliberately isolated the nutrient-knowledge component of the task. We did not test food recognition, portion estimation, meal decomposition, user-entered descriptions, iterative dialogue, or clinician oversight. Errors arising from these additional steps may compound when LLMs are used for complete meals or daily intake assessment. A recent scoping review of language models in nutrition and dietetics found that most published evaluations were conducted in hypothetical or researcher-designed settings and that evidence from real-world implementation remains limited (23). Thus, the present results should not be interpreted as validation of a patient-facing, consumer-facing, or clinician-facing nutrient-assessment tool.

The weaker performance for several micronutrients also has practical relevance. Iron and vitamin A deficiency remain measurable public health concerns in Chinese children and older adults, while consumers and patients may seek nutrition information from generative AI tools (2–4). The present study did not assess user interpretation, trust, dietary behavior, or clinical outcomes and therefore cannot establish downstream harm. However, the component-specific error pattern supports cautious presentation of LLM-generated micronutrient estimates and external verification when accuracy matters, particularly for nutrients with lower agreement or reproducibility. Future systems could incorporate retrieval from validated food-composition databases or provide component-specific reliability information rather than presenting all fifteen dietary components with equivalent apparent certainty (20).

The largest weaknesses were nutrient-specific. Sodium and vitamin C showed consistently high aggregate agreement, whereas potassium, iron, zinc, and especially vitamin A did not. Several nonexclusive explanations are plausible. Micronutrient values may vary with cultivar., animal tissue, fortification, processing, storage, and cooking, and generic food names may correspond to multiple legitimate formulations. Representation in model training data may also differ across foods and nutrients: common energy and macronutrient values are frequently reported in public-facing nutrition resources, whereas some micronutrient values are sparser, more source-dependent, or expressed in heterogeneous units. Macronutrients are themselves variable, but their broader representation and relationships with total energy may provide stronger constraints for plausible estimates. These mechanisms were not directly tested and should therefore be regarded as hypotheses rather than demonstrated explanations.

Percentage-based errors require similar caution. Reference values equal to zero were excluded from percentage-error calculations because percentage error is undefined at zero, but small positive denominators remained in the primary analysis and could inflate MAPE. This was especially relevant for dietary components with many zero or near-zero CFCT values. In the denominator-sensitivity analysis, trimming the lowest 5% of positive reference values reduced mean MAPE substantially for several components, including sodium and vitamin A, while the leading model-ranking pattern was largely preserved. This supports interpreting MAPE alongside ICC, MAE, RMSE, and Bland–Altman analysis rather than treating any single percentage-based metric as definitive.

CFCT should also be understood as the benchmark reference used in this study, not as an error-free biological ground truth. National food-composition tables report standardized values that may not capture regional recipes, brands, seasonal variation, cultivar differences, household preparation, or all analytical uncertainty. Consequently, a model-CFCT discrepancy can reflect model error, a different interpretation of the food label, or genuine compositional variation. Human expert adjudication was outside the scope of this computational benchmark, but future studies could incorporate dietitians or food-composition specialists to distinguish plausible cultural or formulation differences from translation errors, semantic mismatches, or model hallucinations.

Model-level rankings should likewise be interpreted narrowly. GLM retained the leading overall position when the three-run median replaced the mean and when percentage-error handling was varied, supporting the robustness of the broad finding that it performed favorably in this benchmark. However, the exact ordering of lower-ranked models changed under some alternative ranking specifications. The composite score assigns equal weight to metrics with different statistical properties and clinical meanings; it is therefore a descriptive summary of the selected models, prompts, API settings, dietary components, reference dataset, and scoring rules, not a safety index or a durable hierarchy of model quality. Rankings may change with model updates, languages, prompts, retrieval tools, or alternative weighting schemes.

Repeated querying added information that a single-response evaluation would have missed. Energy, fat, carbohydrate, protein, and sodium were highly reproducible, whereas vitamin A was markedly less stable. The mean-versus-median sensitivity analysis left the overall ordering of all nine models unchanged, although a small number of model-component combinations crossed prespecified ICC categories, again concentrated largely among micronutrients. Three runs nevertheless provide only a limited characterization of stochastic behavior. Future benchmarks should use more repetitions when feasible and report both central tendency and dispersion rather than relying on a single generated value.

### 4.1 Strengths and limitations

Strengths of the study include the diverse Chinese food set, inclusion of both Chinese-developed and international models, a common per-100-g basis, multiple complementary agreement and error measures, explicit quality control, and three independent API runs. Linking standardized query names to CFCT entries through predefined food identifiers reduced ambiguity in reference matching, and the final analytic dataset contained complete outputs for all model-run-food combinations.

Several limitations remain. First, differences in API routing, hidden system instructions, model snapshots, and the Kimi temperature constraint may have affected comparability across models. Second, the structured English-language batch prompt does not reflect how lay users, dietitians, or clinicians typically phrase questions, and Chinese-language prompting was not tested. Third, retaining the same food order across runs prevents separating food-specific knowledge errors from positional effects. Fourth, excluding human input and expert adjudication isolates model behavior but limits external validity. Fifth, CFCT values are standardized references rather than absolute truth, and only single 100-g foods—not portions, mixed meals, or complete recalls—were assessed. Sixth, uneven food-group sizes (4–45 foods) make subgroup findings descriptive, especially for small groups. Finally, only three repetitions were used and composite rankings were exploratory, so the findings apply to the tested model snapshots rather than to model families indefinitely.

### 4.2 Conclusion

Under controlled text-based, per-100-g benchmark conditions, general-purpose LLMs showed high aggregate agreement with CFCT values for energy and several macronutrients, but accuracy and reproducibility were less consistent for important micronutrients, particularly vitamin A, zinc, iron, and potassium. Repeated-run and sensitivity analyses showed that the main model-level pattern was robust, while also demonstrating that stability, aggregate agreement, and individual-food accuracy are distinct properties. These findings do not establish clinical equivalence or validate ungrounded LLM estimates for patient care, dietary prescription, or other high-stakes decisions. CFCT should be regarded as the reference benchmark used here rather than an absolute ground truth; when accurate values are required, LLM-generated estimates should be verified against validated food-composition resources and, for clinical decisions, reviewed by qualified nutrition professionals. Future studies should evaluate natural lay and professional prompts, Chinese-language and region-specific descriptions, randomized or counterbalanced food ordering, larger numbers of repeated runs, human expert adjudication, meal- and portion-level inputs, and retrieval-augmented systems linked to validated nutrition databases.

## Data Availability

Model-generated nutrient estimates, derived analytic datasets, and statistical code are available from the corresponding author upon reasonable request. Reference nutrient values from the Chinese Food Composition Table, 6th edition, are subject to copyright and publication restrictions and cannot be redistributed in full.
