# Supplementary material for: Text-based nutrient estimation by large language models: a comparative evaluation against the Chinese Food Composition Table
Source: Front Nutr. 2026 Oct 5;13:1952757. doi: 10.3389/fnut.2026.1952757 (PMC13677351; doi:10.3389/fnut.2026.1952757)
Supplement: Supplementary file 1 [file Table_1.DOCX]

**Supplementary Materials**

**Text-Based Nutrient Estimation by Large Language Models: A Comparative Evaluation Against the Chinese Food Composition Table**

# Supplementary Methods

The final run-level dataset comprised 8,073 observations (299 foods × 9 models × 3 independent runs). Each run was an independent API run with no conversational state carried over. Quality-control checks assessed row and food counts, duplicate model-run-food keys, food-name matching, and missing model or reference values. Food-name values were matched exactly to the prespecified standardized query names; no string normalization, case-folding, whitespace trimming, fuzzy matching, or manual name correction was applied to food-name values.

Two sensitivity analyses addressed reviewer concerns about aggregation and percentage-error metrics. First, accuracy metrics were recalculated using the median rather than the mean of the three runs for each model-food-component combination. Second, the influence of small denominators on MAPE was explored by excluding positive CFCT reference values at or below the component-specific 5th percentile and recalculating MAPE. Composite ranking robustness was further examined by substituting median absolute percentage error for MAPE, omitting the percentage-error criterion, and using the near-zero-trimmed MAPE. All sensitivity analyses were exploratory and were used to assess robustness of the primary conclusions rather than to redefine the primary estimand.

**Supplementary Table S1. Food-group composition of the final 299-food sample.**

| **Food group** | **n** | **% of sample** |
| --- | --- | --- |
| Vegetables and vegetable products | 45 | 15.1 |
| Cereals and cereal products | 35 | 11.7 |
| Livestock meat and products | 30 | 10.0 |
| Fruits and fruit products | 30 | 10.0 |
| Fish, shrimp, crab, and shellfish | 25 | 8.4 |
| Condiments | 17 | 5.7 |
| Dried legumes and products | 15 | 5.0 |
| Snacks and sweet pastries | 15 | 5.0 |
| Tubers, starches, and products | 12 | 4.0 |
| Poultry meat and products | 12 | 4.0 |
| Milk and dairy products | 10 | 3.3 |
| Beverages | 10 | 3.3 |
| Nuts and seeds | 8 | 2.7 |
| Fungi and algae | 8 | 2.7 |
| Instant foods | 8 | 2.7 |
| Eggs and egg products | 8 | 2.7 |
| Fats and oils | 7 | 2.3 |
| Sugars, preserved fruits, candied fruits, and honey | 4 | 1.3 |

Note. Food-group sizes ranged from 4 to 45. Group-level performance analyses were descriptive because subgroup sizes and dietary-component distributions differed.

**Supplementary Table S2. Model-specific quality control and final run completeness.**

| **Model** | **Runs** | **Final rows** | **Foods/run** | **Final missing cells** | **Validation issue and remediation** |
| --- | --- | --- | --- | --- | --- |
| DeepSeek | 3 | 897 | 299 | 0 | Initial run 2 contained 9 missing energy cells in one batch; the entire run was repeated and the complete replacement was retained. |
| Qwen | 3 | 897 | 299 | 0 | None |
| GLM | 3 | 897 | 299 | 0 | None |
| Minimax | 3 | 897 | 299 | 0 | None |
| Kimi | 3 | 897 | 299 | 0 | None |
| Claude | 3 | 897 | 299 | 0 | None |
| Mistral | 3 | 897 | 299 | 0 | None |
| Gemini | 3 | 897 | 299 | 0 | None |
| ChatGPT | 3 | 897 | 299 | 0 | None |

Note. All 27 retained model-run combinations contained 299 foods and complete values for all 15 dietary components. No records were excluded because of food-name matching failure, and no dietary-component value was manually edited.

**Supplementary Table S3. Zero reference values and near-zero denominator sensitivity of MAPE.**

| **Dietary component** | **Reference = 0, n (%)** | **n in primary MAPE** | **5th percentile of positive reference** | **Mean MAPE (%)** | **Trimmed mean MAPE (%)** | **Relative reduction** |
| --- | --- | --- | --- | --- | --- | --- |
| Energy (kcal) | 2 (0.7%) | 297 | 17.8 | 13.4 | 12.6 | 6.0% |
| Protein (g) | 10 (3.3%) | 289 | 0.4 | 26.9 | 17.6 | 34.7% |
| Fat (g) | 9 (3.0%) | 290 | 0.1 | 58.3 | 52.9 | 9.2% |
| Carbohydrate (g) | 26 (8.7%) | 273 | 0.86 | 36.0 | 24.2 | 32.8% |
| Dietary fiber (g) | 144 (48.2%) | 155 | 0.2 | 74.9 | 49.3 | 34.1% |
| Sodium (mg) | 153 (51.2%) | 146 | 3.55 | 242.4 | 106.2 | 56.2% |
| Potassium (mg) | 4 (1.3%) | 295 | 13 | 72.9 | 55.3 | 24.2% |
| Calcium (mg) | 7 (2.3%) | 292 | 4 | 58.5 | 48.3 | 17.4% |
| Iron (mg) | 6 (2.0%) | 293 | 0.2 | 52.2 | 45.7 | 12.5% |
| Zinc (mg) | 13 (4.3%) | 286 | 0.1 | 50.3 | 46.8 | 7.1% |
| Vitamin A (μg RE) | 117 (39.1%) | 182 | 2 | 145.8 | 83.7 | 42.6% |
| Vitamin C (mg) | 204 (68.2%) | 95 | 1 | 83.8 | 65.6 | 21.7% |
| Thiamin (mg) | 31 (10.4%) | 268 | 0.01 | 78.4 | 55.8 | 28.9% |
| Riboflavin (mg) | 22 (7.4%) | 277 | 0.02 | 53.4 | 40.3 | 24.6% |
| Niacin (mg) | 83 (27.8%) | 216 | 0.2 | 53.2 | 38.3 | 27.9% |

Note. Primary MAPE excluded only reference values equal to zero. The exploratory trimmed MAPE additionally excluded positive reference values at or below the component-specific 5th percentile. Because ties at the threshold were excluded, the reduction in sample size could exceed exactly 5%. Mean MAPE values are averages across the nine models with equal model weighting.

**Supplementary Table S4. Model-level sensitivity to mean versus median aggregation of the three runs.**

| **Model** | **Mean accuracy ICC: mean aggregation** | **Mean accuracy ICC: median aggregation** | **Δ ICC** | **Primary rank** | **Median-aggregation rank** |
| --- | --- | --- | --- | --- | --- |
| GLM | 0.906 | 0.904 | -0.002 | 1 | 1 |
| Minimax | 0.852 | 0.852 | -0.000 | 2 | 2 |
| Qwen | 0.818 | 0.826 | +0.008 | 3 | 3 |
| Claude | 0.839 | 0.853 | +0.014 | 4 | 4 |
| DeepSeek | 0.831 | 0.812 | -0.020 | 5 | 5 |
| Gemini | 0.716 | 0.715 | -0.002 | 6 | 6 |
| Mistral | 0.738 | 0.735 | -0.003 | 7 | 7 |
| Kimi | 0.831 | 0.841 | +0.010 | 8 | 8 |
| ChatGPT | 0.711 | 0.706 | -0.006 | 9 | 9 |

Note. Mean accuracy ICC is the arithmetic mean of the 15 dietary-component-specific ICC(A,1) values. Overall model ordering was unchanged when the three-run median replaced the three-run mean.

**Supplementary Table S5. Robustness of the exploratory composite model ranking.**

| **Model** | **Primary rank** | **Median aggregation** | **MdAPE substituted** | **Percentage metric omitted** | **Near-zero-trimmed MAPE** |
| --- | --- | --- | --- | --- | --- |
| GLM | 1 | 1 | 1 | 1 | 1 |
| Minimax | 2 | 2 | 2 | 3 | 2 |
| Qwen | 3 | 3 | 3 | 2 | 3 |
| Claude | 4 | 4 | 4 | 4 | 4 |
| DeepSeek | 5 | 5 | 5 | 7 | 5 |
| Gemini | 6 | 6 | 7 | 5 | 7 |
| Mistral | 7 | 7 | 6 | 6 | 6 |
| Kimi | 8 | 8 | 8 | 8 | 8 |
| ChatGPT | 9 | 9 | 9 | 9 | 9 |

Note. The primary composite used six equally weighted criteria: accuracy ICC(A,1), MAE, RMSE, MAPE, repeated-run ICC(A,1), and median repeated-run CV. GLM remained rank 1 under every alternative specification. Changes below first place demonstrate that the composite ranking should be interpreted as descriptive rather than as a fixed hierarchy.

**Supplementary Table S6. Model-component combinations that changed ICC category under median aggregation.**

| **Model** | **Dietary component** | **ICC: mean** | **Category: mean** | **ICC: median** | **Category: median** |
| --- | --- | --- | --- | --- | --- |
| Minimax | Thiamin (mg) | 0.891 | Good | 0.916 | Excellent |
| Minimax | Vitamin A (μg RE) | 0.504 | Moderate | 0.443 | Poor |
| DeepSeek | Vitamin A (μg RE) | 0.650 | Moderate | 0.467 | Poor |
| GLM | Thiamin (mg) | 0.905 | Excellent | 0.897 | Good |
| GLM | Iron (mg) | 0.910 | Excellent | 0.895 | Good |
| ChatGPT | Vitamin C (mg) | 0.902 | Excellent | 0.843 | Good |

Note. Only 6 of 135 model-component combinations (4.4%) crossed a prespecified ICC category threshold when the median replaced the mean across the three runs. Thresholds: excellent ≥0.90; good 0.75 to <0.90; moderate 0.50 to <0.75; poor <0.50.
